# Anatomical and Physiological Observations Made upon Decapitated Subjects

**Published:** 1869-08-15

**Authors:** Ch. Robin

**Affiliations:** From Robin’s Journal de Physiol, etc.


### CHICAGO MEDICAL JOURNAL.

VOL. XXVI.—AUGUST 1, 1869.—No. 15.

### TRANSLATIONS.

### *Anatomical and Physiological Observations made upon Decapitated Subjects.*

BY M. C. ROBIN.

*From Robin's Journal de Physiol, etc.*

(CONTINUED FROM LAST MONTH.)

**VI—Of Some of the Phenomena of Reflex Action and of Contractility.**

M. Duval observed in two criminals, in 1868, movements by reflex action of the most evident character:

"By *pinching* the skin, and particularly by giving it a sharp blow with the hand, the subjacent muscles were seen immediately to contract; the contraction subsided quite slowly. This experiment has been repeated a great number of times, especially upon the limbs. Amongst other muscles, the contraction of the deltoid, of the biceps brachialis, of the anterior muscles of the thigh, of the gastrocnemii, manifested themselves most clearly.

"In the first subject the reflex power persisted one hour and three-quarters after decapitation. The reflex movements were always more slight and less prompt to develop themselves after the first hour; a more energetic excito-motor blow or a more forcible pinch became necessary.

"In the second subject reflex action was appreciable an hour and a quarter after death.

"The muscles of the neck were, during more than three-quarters of an hour, the seat of fibrillary contractions, very plainly visible of their cut surface, and that without any previous excitation, either mechanical or electric." (M. Duval, *loc. cit.*, p. 522, et *France Medicale*, Août, 1867.)

Dr. Dupri, his students and I, equally, observed in the criminal whom we had under our eyes, the phenomena of contraction of the muscles of the neck, such as those described by M. Duval. In all the muscles of the thorax and abdomen cut for dissection, contractions were produced also many times at more or less protracted intervals after the section, aside from all mechanical or physical excitement, which has moreover been observed in all the animals described soon after death. These contractions are certainly initiated by the novel physical condition of each striated fasciculus, induced by dissection, by the changes of temperature in the portion cut and exposed to the air.

I wish, moreover, to state that when we left the amphitheatre, at 9 o'clock, that is, more than three hours and a quarter after the execution, the fasciculi of the diaphragm cut and still covered by the pleura and peritoneum, still contracted from time to time energetically, almost in a rythmical manner, without physical or mechanical intervention on our part. We determined, in addition, contractions of the muscles of the abdomen and of the arm by pinching the skin, with or without torsion by the aid of the nails or dissecting forceps, the pinching being performed at the level of the muscle in which it was intended to excite contractions, care being taken not to compress nor to drag them. Contractions, inducing still greater prominence of the muscles, were obtained by striking the skin perpendicularly to the direction of the contractile fascicule, either with the edge of the hand or with the back of the scalpel. But this cause of contraction is not of the same order as the pinching. By percussion, in fact, the muscular fibres are acted upon mechanically and directly, in spite of the interposition of the skin of the adipose tissue, and of the subjacent faciae, just as is done when a red muscle exposed is struck. On the contrary, in the case of pinching the skin followed by contraction of the subjacent muscles, the phenomena are upon the trunk of a decapitated criminal, of the same order as the movements by reflex action, properly so-called; that is to say, without the intervention of the direct cerebral acts of sensitive perception and of volition, the impression of the pinching is transmitted to the cells of the posterior horns of the spinal cord, then these by the intervention of their anastomoses with the cells of the anterior cornua placed at the same level, excite the motor activity of these last, which the tubes of the anterior roots transmit to the muscles.

Among the phenomena of this order which we have determined in the experiments above described, the most important is the following, observed about one hour after the execution. The right arm being extended obliquely alongside of the trunk, the hand about twenty-five centimetres outside of hip, I scratched the skin of the chest with the point of a scalpel at the level of the nipple, over an extent of eleven or twelve centimetres without making any pressure upon the subjacent muscles. We soon perceived the pectoralis major, then the biceps, the brachialis anticus perhaps, and the deltoid, contracting successively and rapidly.

The result was a movement of approximation of the whole arm towards the trunk, with rotation of the arm inward, and demi-flexion of the forearm upon the arm, a true movement of defense which projected the hand from the side of the chest towards the cavity of the stomach.

The thumb, which was demi-flexed towards the palm of the hand, and the other fingers demi-flexed upon the thumb, exhibited no movement.

The arm having been replaced in the position which it had previously occupied, we saw it execute a movement like the preceding at the moment when the skin was scratched in the same manner a little below the clavicle.

This experiment succeeded four times, but upon each occasion the movement was less extended; after which the scratching of the skin induced nothing further than a contraction of the pectoralis major, scarcely involving the arm.

We moreover made no further attempts to determine if the innervation exhausted by exercise might be re-established, under these circumstances, after a certain period of repose, as in ordinary experiments upon the nerve, induced by reflex action.

When, after having made the different observations and experiments reported in the preceding paragraphs, we directed our attention to the head, and to the spinal cord, we found all excitability in this latter already extinguished, and we noted equally no movements from pinching the skin of the lips of the cheeks, of the conjunctiva, or from scratching the cornea.

We moreover had no electrical apparatus at our disposal.

The facts set forth in this paragraph only confirm, in man, those which so frequently established in different animals, since Prochaska, Legallois, Marshall, Hall, &c., show that, aside from all intervention of cerebral nervous acts, termed acts of perception and will, an inevitable functional correlation exists between this or that given surface, and the muscles which they cover, in consequence of this fact, that the tubes of the nervous roots, of the same pair, terminate in the same place, at the same level, in same mode, some in the integuments, others in the organs which they cover. If, on the other hand, the configuration and the insertions of the muscles, as well as the arrangement of the surfaces and of the articular ligaments around which they extend, be considered, the fact may be established that the simultaneous and successive contractions of these muscles determine clearly a regular and co-ordinated movement, as well as automatic, and neither anticipated, spontaneous nor instinctive in opposition to what is as yet admitted by many of those who interpret and generalize these actions outside of the data of anatomy and physiology.

Touching the acts accomplished by the nerves brought into play in this case, they consist in a transmission by the tubes termed sensitive of the impression produced by the pinching, &c., as far as the cellules of the posterior cornua of gray matter, which by an act of innervation analogous to those of perception, so-called, but without being identical with them, exciting the activity termed motor of the large cells of the corresponding anterior cornua; motricity transmitted as far as the muscle of the tubes originating from these cells, and which form the net-work of the anterior roots and of the motor nerves.

It is especially these acts of motricity which have been particularly considered as of an intellectual character, inasmuch as the anatomical elements of the gray nervous substance and their connection have not been known.

But they can be accomplished in the cord, and they are independent of cerebral innervation, which, when it intervenes, effects only their excitation or modification in this or that direction, but does not supply them. It only commands without executing them.

The observations reported above agree on the other hand with the experiments of Brown-Sequard, which prove that muscular contractility persists for a much longer time after the cessation of the circulation in men and other large animals than in the smaller mammiferæ (Brown-Sequard *loc. cit.*, 1858 in 8vo, p. 354).

*Condition of the encephalon and of the skin.*—We will add here a few anatomical remarks upon the preceding physiological data.

The skin of the criminal above referred to, submitted to our observation the morning of the fifth of September,

1868, the body being still warm, did not present to us the condition of cutis anserina; the muscles of the lower limbs began to exhibit a slight cadaveric rigidity, when we had left it three hours and a quarter after the execution. The penis was flaccid, not retracted. On the contrary, the two other criminals of whom I have spoken already, observed in the laboratory with MM. Legros, Goujon, etc., the one on the tenth of March, the other on the twenty-eighth of November, 1867, in a sufficiently low temperature, the first six and the second ten hours after the execution, had the skin in the condition of cutis anserina in the highest degree. The corpus cavernosum was retracted and hard, as in a man emerging from a cold bath. The cadaveric rigidity was general without being extreme in the latter.

The preceding facts exhibit very well the influence of cold, and the absence of all sanguineous congestion in the production of furrows and rugosities of the skin, characterizing the preceding state.

In the first of the criminals observed in my laboratory, and who was about twenty years of age (Lemaire), the weight of the brain, the pia-mater and the medulla oblongata included, was 1,183 grammes. It was remarkable by its flatness, by the narrowness of the anterior lobes, which constituted upon the sides of the mass considerable and abrupt ridges.

The pia-mater was remarkable by its extreme tenuity and especially by its adhesion to the gray substance of the convolutions. This adhesion was such that it was impossible for M. Legros to raise up this membrane without dragging with it a portion of the superficial gray substance of the thickness of one or two millimetres. It was necessary to abandon this operation after having raised several square centimetres of this envelope and crushing the encephalon remaining in position.

In the two other subjects the pia-mater, perceptibly thicker, without exceeding the normal thickness, was readily detached from the surface of the convolutions without tearing up their substance.

The encephalon of the one of these who was more than sixty years of age (Davilain or Avilain) weighed 1,277 grammes, the pia-mater included. That of the last criminal, aged about thirty two years (Blanc Gonnet), could not be weighed while still warm. The white and gray substance had a consistence a little more firm than what is ordinarily observed in autopsies. Neither offered anything particular regarding conformation or volume of the encephalon.

In these two latter the walls of the cerebral ventricles and of the fourth ventricle were found in contact, simply moist, with only a few drops of serosity in each of the ancyroid cavities and in the anterior portion of the third ventricle.

**VII—Remarks upon Certain Tissues and Fluids of the Economy Observed in the Recent State.**

In the two criminals whose lungs were observed, the one at six or seven and the other at ten or twelve hours after death, the posterior portion of the lower lobe of these organs had become livid by hypostatic congestion of the capilaries. The other portions of the pulmonary tissue had the grayish color, the marbled aspect, with some black interlobular marks, which are ordinarily observed in the dead body.

In the oldest of the subjects the apices of these two organs were emphysematous, with a little air in the interlobular spaces, especially in the right.

In the last of the criminals the lungs, still warm, presented no hypostasis; they presented the color already indicated. The summits of the two lungs, especially that of the left, were sprinkled with *gray granulations*.

The color and consistence differed in no respect from that which are observed in the lungs of subjects upon whom autopsy is made from twenty-four to forty hours after death.

Near the apex of the left lung there was a corrugated cicatrix and cavity of the size of a nut, filled with pus, surrounded with tissue rich in gray granulations. The consistence and the color of this morbid product, yet warm, did not differ from what is observed in autopsies of analogous cases.

The same was the case with the adhesions existing between the pleura pulmonalis and the pleura costalis.

In the three subjects the spleen was firmly contracted upon itself. Its tissue had nearly the consistence of that of the liver. Its lacerated surface was irregularly granular, and gave only *splenic pulp* when scraped.

The tissue of the liver uniformly of a reddish brown, without distinction of two substances—the one red, the other yellow—exhibited as granulated fracture and the consistence habitually observed in autopsies.

In the subject whose abdomen was opened one hour after death, and whose viscera were yet warm, more peristaltic contraction of the intestines was observed even after pinching. The abdominal viscera exhaled no intestinal odor, but only a slight odor of stale meat.

In the subject whose abdomen was opened seven hours after death, the organs exhaled a slight odor usually termed intestinal. It was likewise apparent, but decidedly more marked, in the one whose abdomen was opened twelve hours after the execution. In this last, the organs contiguous to the fundus of the gall-bladder were tinged slightly greenish, whilst they were not at all so in the two others.

The color of muscular coat of the intestine was a pale, semi-transparent gray, slightly reddish, and that of the bladder was, on the contrary, of a pale, semi-transparent gray, slightly bleached; this color, in fact, did not differ sensibly from that observed in subjects submitted to autopsy, in which these tissues have undergone no morbid alteration. *Intestinal Mucus*—In two of the subjects the mucus and the intestinal contents, examined by MM. Goujon,

Legros, and myself, gave an acid reaction from the cardia to the rectum inclusive. The acidity, very marked throughout the entire stomach, was on the contrary very feeble in the duodenum and the cæcum, and then diminished progressively throughout the remainder of the large intestine. These two subjects had died during the digestive process, the jejunum and the cæcum alone, each contained several cubic centimetres of gas.

In two of the subjects the contents of the small intestine, as far as the middle of the ileum, were of a creamy consistence, of a whitish gray color; it had, indeed, all the characteristics attributed to chyme in the descriptions of authors.

In the other subject, who had eaten quite a large quantity of green leguminous food, the chymous paste had preserved this color, and exhibited a consistence a little more pasty than in the preceding. The prismatic cells of the epithelium of the small intestine were filled with fatty granulations, which rendered them opaque.

In these, as in the mucus of the small intestine, and often also in that of the stomach, there are found in all subjects dead from disease, observed from twenty-four to forty-eight hours after death, numerous short and rigid filaments of *Leptothrix* (bacteria). Sometimes, also, Vibriones or *Spirillum* are seen. Now, in the first of the subjects the examination of the mucus of the duodenum, and of the rest of the small intestine, made about eight hours after the execution, showed none of these filaments nor any vibriones. In the second the examination, made about thirteen hours after death, showed a small number of *Leptothrix* only, and no vibriones.

### BILE.

In three subjects the gall-bladder, almost full, contained about thirty grammes of bile. In one of them, collected while warm, it had a stale, slightly nauseating odor, but without any other well-defined characteristics. It presented the same characters, to a little less marked degree, in the two other subjects studied in this point of view nine hours and twenty-eight hours after death.

Whilst very mobile and fluid this liquid, in the three subjects was very ropy when it was poured from a height, or when the finger was applied to it and then withdrawn. This last characteristic disappeared after ebulition.

This bile did not change, sensibly, either blue or red litmus paper; whilst at the end of a quarter of an hour of contact the red litmus paper had resume, to a slight degree, its bluish color.

Before and after ebulition it had, to a very decided degree, the bitterness peculiar to bile, the taste which is imparted to it by the tauro-cholate or choleate of soda.*

Its color was a fulvous brown, with greenish, semi-transparent reflection in one of the subjects; in the second it was semi-transparent, of a pale fulvous yellow, not greenish; lastly, in the third, examined scarcely two hours after death, it was remarkable for its deep orange yellow tint, broken by some small clots of the same tint, but nearly opaque, and it has communicated this same tint around the pancreatico-duodinal orifice, over a surface equal in size to that of a five-franc piece.

These facts are important to be noted because they show, with others, that bile, normally secreted, can present not only very great variations of tint (voyes Ch. Robin, *Leçons sur les Humeurs*, 1867, p. 537), but even with varying proportions of its coloring matter to the degree of being almost colorless, if only a few drops be observed at a time.

Moreover, may we not consider as demonstrating that the bile is colored more and more deeply during its retention in the gall-bladder, the fact cited by Aran, who observed bile transparent and scarcely colored issuing from a

---

* It is well known that Sassaigne has determined the absence of picromel (impure cholate or choliate) in the bile of the fœtus of the cow at six months, with chloride of sodium of carbonate of soda, and of phosphate of lime recognized in the meconium. (Annales de Physique et de Chimic, Paris, t. 17, p. 304; 1821.)

trocar (canula ?) plunged, by mistake, into the substance of the liver (See Liegois, art. Bile, du Dict. Encyclopédeque de Médicine, Paris 1868, t. IX, p. 267.)

Its color did not change by exposure to the air, before, as after ebulition.

Examined with the microscope, before the action of heat, the bile exhibited only prismatic epithelial cells, without vibratile cilia, as those of the mucus surface of the gall-bladder, some isolated other juxtaposed to the number of from two to ten or thereabouts. Left at rest it deposited at the bottom of the vessel a slight turbid deposit, composed of cells and little microscopic flocenti of mucus. It exhibited neither *leptothrix* (Cacteria) nor vibriones which are contained in the bile of nearly all dead bodies at the time of examination. It exhibited aslo some few minute drops of fat. The little clots or flocnti which it held in suspension were composed of semi-fluid mucus interspersed with grayish granulations, agglutinating some epithelial cells, and some fatty drops, and becoming striated in contact with acetic acid.

Moreover, acetic acid poured upon fresh bile caused it instantaneously to be converted, as is seen habitually, into a yellow mass, forming filaments, the flocculi adhering one to another; the substance of these latter appearing granular and striated under the microscope; it contained many spherical yellow drops, frequently voluminous, of an oily or resinous aspect. At length these floculi were deposited at the bottom of the test tube, and were no longer easily mingled with the remainder of the liquid by agitation. Sulphuric acid acted at first like the former reagent, but rapidly changed the mass into a reddish brown. Nitric acid rendered the bile thick, very much clotted, then successively greenish, of a blackish green, reddish, then finally left it grayish and decolorized. The same effects are produced by these acids upon the previously boiled, whilst the liquid is then a little less flocculent. Fresh bile from dogs exhibited the same effects under these reagents, but it remained violet after the action of nitric acid.

The bile of these two subjects and the bile of a dog recently killed examined comparatively, boiled, became of a greenish-brown, and there were developed little grayish clots sparsely floating in the liquid, depositing themselves at the bottom of the test tube in a thick stratum of several millimetres in a depth of liquid of seven or eight centimetres. Although changed to a deeper tint, the supernatural liquid remained more limpid than before, having no longer the slightly turbid appearance which it formerly exhibited when it was examined by transmitted light. Moreover, it ceased to be filamentous, as before.

The little clots, formed under the influence of heat, were friable, easy to be separated formed of a finely granular non striated substance, and enclosing a few fatty granular anp broups of prismatic epithelial cells.

It will be perceived from the preceding that it is not perfectly correct to say with some authors (See Vulpian, *le Foie et la Bile*, *Reone*, *des Cours Scientifiques*, Paris, 1867, p. 46) that the bile coagulates by heat, for that which coagulates by heat is the mucus superadded to the bile by the mucous membrane of the gall-bladder, and only in very small quantity (See C. Robin *Leçons sur les Humeurs*, 1867, p. 473 and 547). Schultz, Berzelius, Dumas and many other observers have moreover asserted, for a long time, that bile does not coagulate by heat.

Besides, science demands still that a comparative examination be made in the preceding regard, between the bile flowing from the hepatic duct, before its arrival at the gall-bladder, and that which has already remained in that receptacle.

Acetic and nitric acids likewise coagulate this mucus, but beside, they precipitate the tauro-cholic acid in the state of little resinous drops. As to the coloring matter, they attack it likewise; but it cannot be determined if this action is such as will permit bile to be classed among the liquids coagulable by acids; for it is not yet known whether they cause it to undergo a true coagulation, after the manner of that which they produce in casein, for example.

GENITO-URINARY ORGANS AND SEMEN.

In one of the subjects the bladder, the seminal vescicles and the prostate were removed in examining the rectum, about two hours after the execution.

A primary fact attracted our attention in this subject: the absence of all urinous odor, or of that usually termed intestinal in these organs, and in the surrounding lammillar tissue, on the contrary to what is observed in these parties in all autopsies, from twenty-four to forty-eight hours after death. Thirty hours later, notwithstanding the retention of these tissues in a jar, in a temperature of twenty to twenty-five degrees (centigrade), they had assumed no cadaveric, urinous nor fæcal odor. It had still only the faint odor of fresh tissue.

In the second place, we, with M. Dupre and his students, remarked the absence of all odor in the semen expressed from the cut vasa deferentia, and from the opened seminal vescicle, even after rubbing the liquid between the fingers. The milky fluid expressed from the prostate gland upon the side of vera montanum presented to us the same absence of all odor either spermati, intestinal or fæcal. Thirty hours later this liquid was yet inodorous.

It is after observations of this sort, made upon men and animals, that I have detected that, taken isolated, the fluids which unite to constitute the semen do not exhale the peculiar spermatic odor, and that this is not developed except at the instant of ejaculation (*loc. cit.*, 1867, p. 361).

Without observing what might be the odor of the contents of the seminal vescicles in the subjects examined by him, M. Duval asserts that he has discovered a very different odor from that which is exhaled during life. (M. Duval Congress Médicale, 1867, p. 527.)

The semen expressed from the divided vasa deferentia was thick, of a creamy consistence. That of the seminal vescicles was heavier than water, of the consistence of jelly, of a yellowish gray color, semi-transparent, neither lactescent nor opalescent. It was clotted under the finger; neither viscous nor filamentous.

Six hours after, microscopic examination demonstrated that this clotted condition was due to the presence of sympexia, many of which were more than the tenth of a millimetre in thickness, agglutinated together, and containing encysted spermatozoa.

The free spermatozoa moved actively. Thirty hours later they were motionless, and the semen had lost its gelatinous consistence, and its clotted condition to become diffluent and less transparent than before. The *sympexia*, especially, were not liquified; but they were no longer agglutinated together.

The prostrate liquid, examined at the moment of the autopsy, and several hours afterward, was of a milky color, quite fluid, composed of a colorless fluid, holding in suspension fine granulations and fatty globules, some prismatic epithelial cells and some hyaline drops of a viscous consistence.

The urine contained in the bladder was clear and inodorous.

In the two other subjects observed from six to seven hours and twelve hours after execution, the semen of the vasa deferentia was semi-fluid, of an opaque, creamy, slightly yellowish white color. That of the seminal vescicles was semi-transparent, grayish in the younger, of a yellowish white in the elder; it was neither viscous nor filamentous, nor granular; it was free from *sympexia* in one of these, and presented a few in the other; it was free from all spermatic and had only a urinous or slightly fæcal odor.

This fact should be associated with the absence of all odor of this kind in the cellular tissue of the fundus of the bladder, in the surroundings of the rectum, and in the seminal vescicles, in the subjects whom M. Dupra, his students, and myself have examined in this regard about two hours after death. It shows that the fæcal or urinous odor of these parts, and of the semen of all subjects examined from twenty-four to forty-eight hours after death (see Dieu *recherches sur le sperm de veillards, Journal de l'Anat. et de la Physiol.* in 8vo, p. 462), is due to the gradual imbibition of the odorous principles of the urine and the fæces, and that it does not exist before this imbibition.

Thirty-six hours after death all the spermatozoa were motionless; but by warming a portion up to a temperature of forty degrees (centigrade) we saw one or two per eent. of the spermatozoa resume feeble movements, although sufficiently energetic to determine their progression.

In the three subjects, the urethral mucus contained a few spermatozoa, free epithelial nuclei, regularly polygonal cells, with one or two voluminous nuclei, large flattened polygonal cells of the size of those of the œsophagus. Some of these different cells were hollowed with hyaline excavations.

The movements of the vibratile cilia of the epithelial cells of the trachea were still very energetic thirty hours after the execution.

### PERICARDIAC AND VAGINAL SERUM.

In the three subjects the internal surface of the pleura and of the peritoneum was slightly humid, but contained no liqnid, notwithstanding the presence of pleuritic adhesions, and of gray pulmonary granulations in one of them. The pericardium of one of them was in the same condition, as were the tunicæ vaginales in the two younger. The pericardium of the subject aged twenty years contained about thirty grammes of serum as limpid as water, without viscosity, semi-transparent, of a citron yellow and neutral. It became white and milky, without solidifying, by the application of heat.

This liquid, collected twelve or thirteen hours after death, left at rest, became, all at once, transparent, and yielded a thin layer of grayish deposit. This last was composed of quite large flakes of tessalated epithelial cells rolled up or flattened, resulting from the desquamation of the epithelium of the serous membrane. There were, also, isolated cells. Of these different cells isolated or aggregated into flakes, some were very small, finely granular, grayish; others were five or six times larger than the preceding, non-granular and transparent, with large, pale, oval nuclei.

This subject had a small hydrocele on the right side, formed by a neutral liquid, of a citron color, limpid, coagulating by heat.

The kidneys of the same subject contained each two or three cysts of the size of a nut, filled with a colorless liquid, clear and limpid as water, and neutral, and which was rendered white and opaque by heat and nitric acid, without being coagulated.

